# Latent Abnormal Pathology Affects Long-Term Graft Function in Elder Living Renal Allograft Recipients

**DOI:** 10.1155/2013/605704

**Published:** 2013-09-19

**Authors:** Linlin Ma, Lei Zhang, Yu Du, Zelin Xie, Yawang Tang, Jun Lin, Wen Sun, Hongbo Guo, Rumei Bi, Mengmeng Zhang, Xi Zhu, Ye Tian

**Affiliations:** Urology Surgery Department, Beijing Friendship Hospital-Affiliated Capital Medical University, Beijing 100050, China

## Abstract

*Objective*. This study evaluated the long-term effects and clinical significance of latent abnormal pathology on elder living donor kidney graft function after renal transplantation in China. *Methods*. One-hundred and thirty-eight living donor renal transplantations have been carried out at our hospital in recent years. Of these, 72 Time-Zero biopsies were performed and used in this analysis. Clinical data were retrospectively measured at 3, 6, 12, and 24 months after renal transplants. Relationships and effects from biopsy results taken from implanted donor kidney grafts were analyzed. *Results*. Time-Zero biopsy pathology results from donor kidneys showed that 48.61% of donor kidneys had latent abnormal changes; arterial lesions of donor kidneys had significant effects on the renal function of grafts after 2 years' transplantation; correlations between donor age and arterial lesions were significant; and Time-Zero biopsy pathology results could help predict the long-term function of a renal graft. *Conclusions*. Existing latent pathological changes of an elder living donor kidney before transplantation could affect long-term renal function. Whether a senior donor is used should be very carefully considered.

## 1. Introduction

The shortage of organ donors presents a major obstacle for adequate treatment of patients with end-stage renal disease. Living-related kidney donor is turning to be an important organ resource for transplantation. In China, this problem is even more serious. As “the one family one child” family plan has been carried out for 30 years, most of the young generations do not have brothers or sisters. Parents are one of the most possibilities to be a donor. Thus more and more elder donor and marginal living-related kidneys have been received in China in recent years. Whether the quality of kidneys from these sources has an effect on the long-term survival of renal grafts and to what extent has become a focus of concern.

“Time-Zero biopsy” [1] or “baseline biopsy” [2] pathological examinations are conventionally carried out before renal transplants. They have become an important way to detect donor kidney status and to investigate renal graft function in long term. Many centers have included donor kidney biopsy pathological examinations into their routine diagnosis and treatment regimens. Research has suggested that Time-Zero biopsies can identify pathological changes to donor kidneys that cannot be observed by routine noninvasive examinations [3]. Moreover, some pathological changes identified by a kidney Time-Zero biopsy may be related to a recipient's long-term renal function after transplantation. There were a few centers summarizing the experience on the relationship with the latent pathologic changes of Time-Zero biopsy and long-term function of the living donor kidney in China although this examination has been carried out by some transplantation centers in China. This paper observed the long-term results of renal function from 72 living donor renal transplantation cases that have received Time-Zero biopsy at our center and analyzed the impact of existing latent pathological changes to donor kidney grafts on long-term renal function.

## 2. Material and Methods

### 2.1. Study Population

Patients who had received a renal transplant operation from a living donor and had undergone a donor kidney Time-Zero biopsy at our department between November 2007 and November 2008 were examined. All donors were strictly screened using a comprehensive physical examination before donation, including routine biochemistry, blood, urine, stool examinations, infectious disease screening, radioactive isotope renography glomerular filtration rate, renal artery computerized tomography angiography, abdomen type-B ultrasound, X-ray chest examination, and electrocardiography. Donor kidney criteria were in accordance with living donor kidney donation recommended principles of the Transplantation Society, Live Donor Kidney Transplantation Amsterdam Forum, 2004 [4], and the *Clinical Practice Guidelines of Living-Related Donor Kidney Transplantation* [5]. Both donors and recipients were required to sign kidney donation volunteer forms and informed consent forms. Donation and transplantation operations were approved by the ethics committee at our hospital.

### 2.2. Time-Zero Biopsy Method of Renal Graft

Donor nephrectomy approaches were all retroperitoneal open-loop nephrectomy. After dissection of the kidney and before the interrupted renal pedicle, a biopsy of the inferior pole of the kidney was performed with a 16 G bard biopsy needle. Biopsy specimens were put into ice cold saline (0°C) for 30–45 min before tests. Pathological examination preparation involved routine paraffin embedding and sectioning prior to hematoxylin and eosin and immunochemical staining. Sections were observed with microscopy. Diagnosis of pathological abnormalities complied with relevant criteria of urinary disease in *Diagnostic Pathology* [6]. Pathological examinations were performed at the Renal Pathology Laboratory of the Nephrology Department at the Affiliated Beijing Friendship Hospital of Capital Medical University.

### 2.3. Clinical Research Methods

According to donor kidney biopsy pathological results, donor kidneys were grouped into an abnormal group and a normal group. Cases with pathological abnormalities were subdivided further according to the pathological type. Relationships were observed between recipient renal function and different pathological abnormalities of the donor kidneys and the result of long-term recipient renal function. Renal function evaluation parameter in clinical examination data of participants were collected at 3, 6, 12, and 24 months after transplants.

Exclusion criteria were (1) to avoid any influence on pathological observations, occurrence of severe acute rejection reaction without reversion during the perioperative period; (2) cases without recovery of renal function or primary renal graft dysfunction; (3) severe infection or other complications that led to irreversible renal dysfunction; and (4) death within the observation period.

### 2.4. Renal Function Evaluation Criteria

Serum creatinine (serum Cr), blood urine nitrogen (BUN), and blood uric acid were tested using a UniCel DxC 800 system (Beckman Coulter, Los Angeles, CA, USA). Normal ranges for biochemistry testing are taken from *Laboratory Diagnosis* [7]. After quality control analysis, ranges were adjusted to serum Cr, 60~115 *μ*mol/L; BUN, 3.5~6.7 mmol/L; and blood uric acid, 350~440 mmol/L.

### 2.5. Statistical Analysis

Data were evaluated using SPSS 13.5 statistical software. Data are presented as arithmetic mean values ± SD. Comparative analysis of histological and pathological changes and clinical data between groups was carried out using *t*-tests. A *P* value of less than 0.05 was considered statistically significant.

## 3. Results

### 3.1. Clinical Demographic Data

A total of 138 patients with uremia received living-related donor kidney transplants between November 2007 and November 2008. One-hundred and twenty donor kidneys underwent a Time-Zero biopsy. Three patients died within 1 month after transplantation; one patient succumbed to alimentary tract hemorrhaging, and two to cerebral hemorrhages. Two years after transplantation, 21 cases (17.5%) were lost to followups. Seventy-two patients were periodically monitored with followups, with complete data recording and qualified pathological specimens taken in accordance with observation criteria, and were enrolled in retrospective analysis.

Seventy-two living-related kidney donors were qualified according to physical examinations without contraindications or other illness histories that might affect renal function. Of the 72 recipients, primary diseases included primary purpura nephritis (one case), diabetic nephropathy (two cases), and chronic glomerulonephritis and uremia in all other cases. Because of limited medical and economic conditions, only five cases received biopsy examinations of their kidneys and conformed to the diagnostic criteria for chronic glomerulonephritis. Two patients received a second renal transplantation. Donor-recipient gender mismatches occurred in 45 cases. Parents donated a kidney to their child in 28 cases; siblings donated in 18 cases; couples donated in four cases; a nonlineal senior donated in 10 cases; and cousins donated in 12 cases. There were no significant differences observed in the body mass index of donors and recipients, and there were no significant differences observed in the choice immunosuppresive agent between with and without latent pathological exchanges in donated graft. Detailed demographic data are presented in Table 1.

### 3.2. Immunosuppressive Therapy

All patients received a postoperative routine triple immunosuppressive maintenance regimen after transplantation. Thirty-seven patients received a combination regimen of tacrolimus (FK506; initial dose: 0.1 mg/kg/day), mycophenolate mofetil (initial dose: 1.5 g/day), and prednisone (initial dose: 10 mg/day). Thirty-five patients received a regimen of cyclosporin A (initial dose: 6 mg/kg/day), mycophenolate mofetil (initial dose: 1.5 g/day), and prednisone (initial dose: 10 mg/day). Tacrolimus and cyclosporin A doses were adjusted according to plasma drug concentrations. Antithymocyte globulin induction therapy was carried out in 58 patients at a dosage of 0.75~1 mg/kg/day for 3–5 days. One patient received induction therapy using basiliximab at day 0 and day 4 after transplantation. No other patient received induced therapy. Among all 72 cases, there was no occurrence of irreversible renal dysfunction caused by severe complications during the perioperative period.

### 3.3. Pathological Examination

Of the original 120 biopsy specimens, instances of death, missed followups, and disqualified specimens (in cases where less than seven glomeruli were defined, as per prior criteria) reduced the final specimen count to 72. These specimens were qualified by meeting all criteria and were enrolled in the study.

**Figure 1:**
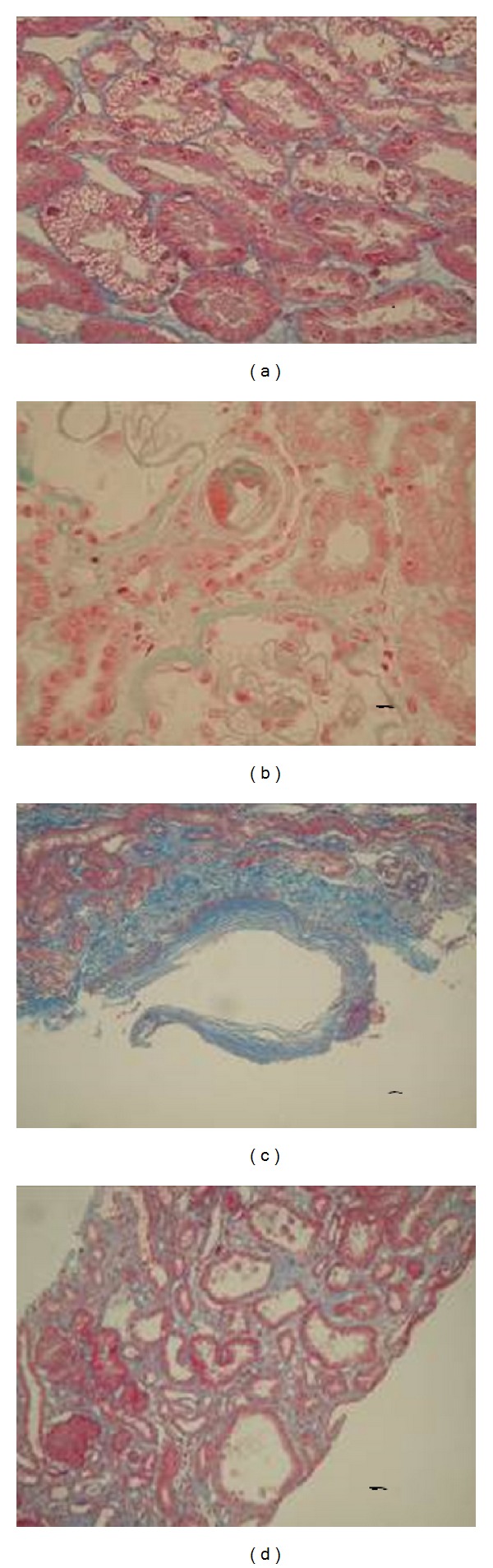
Discovering the latent pathological changes in elder donor kidney by Time-Zero Renal Biopsy. (a) Tubular cavity degeneration. (b) Capillary arteriolar pathological changes. (c) Intimal thickening and arteriolosclerosis. (d) Focal interstitial fibrosis.

Of the 72 donor kidney Time-Zero biopsies, 37 cases were without abnormal pathological change and 35 cases had pathological changes to a varying extent and type; the abnormal rate was 48.61%. Multiple abnormal pathological changes existed on one patient's biopsy sample in partial patients. Abnormal pathological changes were observed in glomeruli, tubules, capillaries, arterioles, and the renal interstitium of nephrons (Figures 1(a)–1(d)). The main issues were (1) glomerulosclerosis: 1/3–1/14 of total glomeruli of biopsy specimens had glomerulosclerosis; (2) renal tubular injury: there were losses in the tubule epithelial brush border, slight vacuolar degeneration of the tubular epithelium, tubular ectasia, granular degeneration, and focal tubular atrophy; (3) capillary and arteriole change: slight capillary arteriolar hyalinosis, intimal thickening, and arteriolosclerosis; and (4) focal renal interstitial fibrosis. Data of the various pathological changes are shown in Table 2.

### 3.4. Relationships between Donor Kidney Pathological Abnormalities and Postoperative Renal Functions of Recipients

Of the 72 cases, there were statistically significant differences in age between those with or without pathological changes (*P* = 0.02). There were no significant differences in total renal function, regardless of whether viewed over the short or long term (Table 3). Of the various pathological changes in donor kidneys, the most noticeable were arterial lesions contributing to renal function deterioration in the long term; a statistically significant observation (Table 4).

### 3.5. Relationships between Donor Kidney Pathological Changes and Recipient Renal Functions

(i) Time-Zero biopsy results showed 22 cases with renal tubule lesions, which included epithelial vacuolation, loss of brush border, slight renal tubular ectasia, and focal renal tubular atrophy. When compared with 50 cases that did not show any tubule lesions, serum Cr concentrations in the tubule lesion cases showed an increase after 2 years (tubule lesions: 156 umol/L; no tubule lesions: 128 umol/L). After excluding the effects of all arteriolopathy and microangiopathy, there were 17 cases with simple tubule injuries, with a mean serum Cr concentration at 2 years after transplantation of 115 umol/L. The mean serum Cr concentration at 2 years after transplantation was 111 umol/L in 41 cases in which there were no tubule injuries and no arteriolopathy and microangiopathy. Differences in serum Cr concentrations between the two groups at 2 years after transplantation were not significant. 

Loss of brush border in renal tubule injury represented typical ischemic damage. Of these cases, puncture biopsy was performed before there was any circulation interruption; therefore, changes were considered to be related to biopsy sampling, preservation, and shipment processes rather than as a result of any renal pathological change. It is of note that the mean age of donors with renal tubule pathological changes was more than that of donor without the same condition, although the difference was statistically significant (44 years versus 37 years, resp.; *P* = 0.045). It is possible that at the same condition, an increase in the age of a donor may be related to a worsened tolerance to ischemic damage.

(ii) Fifteen cases received a donor kidney that developed focal interstitial fibrosis. The mean serum Cr concentration at 2 years after transplantation was 154 umol/L. Compared with the 50 cases in which there was no such pathological change (serum Cr: 125 umol/L), there was no difference in renal function 2 years after transplantation. When patients with and without fibrosis were compared, a statistically significant difference in ages between the two groups was observed (46 years versus 37 years, resp.; *P* < 0.05). It is therefore suggested that focal renal fibrosis changes could be a sign of renal physiological degeneration.

(iii) It is possible that latent vascular lesions in donor kidneys could be the main reason affecting long-term renal function of recipients. There were significant differences in renal function data of serum Cr (*P* < 0.05) and BUN (*P* < 0.05) concentrations between recipients who received renal grafts with or without latent arteriolopathy and microangiopathy after transplanted operation. There were also significant differences between the ages of donors in these two groups. Those donors with latent pathological changes were older than those donors without such changes (*P* = 0.001). Deteriorating renal function was more serious in cases in which the patient had higher grades of pathological change in arteriolopathy and microangiopathy. 

(iv) Sclerosis changes of glomeruli involving capillaries could affect renal function directly. In the current study, there were four cases with renal glomerulosclerosis. The mean age of the four donors was 54 years, which is 14 years older than those donors without glomerulosclerosis. Serum Cr concentrations were also significantly higher in those patients with glomerulosclerosis compared with those patients without glomerulosclerosis after transplanted operation. It is therefore suggested that donor kidneys with arteriolopathy and microangiopathy can lead to abnormal long-term renal function in recipients. These two pathological changes were significantly related to donor age (Table 5). The levels of uric acid were normal when each group was compared with and without pathological changes. This result suggests that deterioration in long-term graft function may be closely related to arteriolopathy and microangiopathy.

## 4. Discussion

To our knowledge, this is the first study investigating the correlation of the latent pathological changes of living donor with two-year graft function in China. The living kidney donors in this study underwent strict preoperative examinations and were a healthy population as qualified by clinical tests. However, Time-Zero biopsy results showed that a considerable number of donor kidneys had latent pathological changes that could not be detected by clinical noninvasive tests, especially the elder donated kidney in our study. Cosio et al. reported that 5% of kidneys in 7% of donors show interstitial fibrosis at the time of kidney donation [8]. Progression of fibrosis in such a kidney could continue for 4 months after a transplant and could progressively affect over 7% of kidneys 2 years after transplantation, with the severity of fibrosis being significantly related to renal graft loss. All kidney interstitial fibrosis in the current study's cohort was slight and sporadic focal fibrosis. It was therefore not a threat to renal function at 2 years after transplantation.

Many researchers studying renal allograft Time-Zero biopsies have reported that latent pathological changes in donor kidneys affect the postoperative renal function of the recipient, and this could help predict long-term survival [9, 10]. A meta-analysis analyzed 16 clinical research reports on renal allograft biopsy in Europe and concluded that glomerulosclerosis, arteriolopathy, and renal interstitial lesions affect long-term survival of a renal graft [11]. The study cited results of 2300 donor kidneys from the United Network for Organ Sharing between 1999 and 2002 by Cicciarelli et al. [12], and a Time-Zero biopsy was conducted in 25% of cases. The conclusion was that the severity of sclerosis of renal glomeruli was significantly related to the survival of a renal graft, delayed graft function, and primary renal dysfunction (*P* < 0.001). Other research has also indicated that if the extent of glomerulosclerosis of a donor kidney is greater than 10%, long-term survival of the renal graft decreases significantly. Escofet et al. reported that every 1% increase in donor kidney glomerulosclerosis results in a 0.8 mL/min decrease in glomerular filtration rate 4 years after transplantation [13]. Each single acute rejection episode would lead to a 7.5 mL/min decrease in the glomerular filtration rate. Ten percent glomerulosclerosis would be equal to the pathological damage of a single acute rejection episode.

Results from the present study's cohort accord with the historical literature. There were four donor kidneys with glomerulosclerosis and a degree range in 7.69–33.33% (mean: 17.02%). Long-term renal function in these cases was significantly lower than that in those cases without glomerulosclerosis. Of all the pathological changes, glomerulosclerosis has the most profound effect on long-term renal function, and long-term renal function of recipients with glomerulosclerosis was significantly more harmful than that of those recipients without glomerulosclerosis.

Glomerulosclerosis appeared mainly in cases in which a parent or a senior aunt or uncle donated a kidney. In such cases, the donors' ages were much older than the ages of those donors in whom there were no pathological vessel changes. It is therefore suggested that the effect of glomerulosclerosis on long-term renal function is related to a donor's age. In these cases, preoperative physical examination and various test results were normal, but biopsy pathologically verified that the donor kidneys had thickened renal arteriolar walls, capillary hyalinization, and glomerulosclerosis of varying severity.

Lubuska et al. [9] observed various graded pathological changes of living donor kidneys and classified arterial pathological changes into four grades according to the extent of thickening and hyalinization of the arteriolar wall. Grade 0 is defined as no pathological change; Grade 1 as pathological change not exceeding 25%; Grade 2 as pathological change of 25–50%; and Grade 3 as pathological change exceeding 50%. Although cases with a grade higher than Grade 1 were not observed in the current study, a review of the literature suggests that (1) pathological change of arterioles could certainly affect immediate postoperative and long-term renal function and (2) the age of a donor is positively correlated with the occurrence rate and severity of arterial pathological changes. 

Similar to the results of Lubuska et al., in the current study, there were 15 cases with arterial pathological changes. Nine cases were evaluated as Grade 1. The mean age of donors was 46 years. The mean 2-year serum Cr concentration was 196 umol/L for these nine cases. A comparison with those cases that did not show arterial pathological changes showed a significant difference (*P* < 0.05). Six cases were evaluated as Grade 2. The mean age of donors was 53 years. The 2-year mean serum Cr concentration was 227 umol/L for these six cases. A comparison with those cases that did not show arterial pathological changes showed a significant difference (*P* = 0.038). The mean age of donors without arterial pathological changes was 40 years. The mean age of the four cases with glomerulosclerosis in this group was 54 years. The 2-year mean serum Cr concentration was 270 umol/L. Comparison of these four cases with those cases without arterial pathological changes showed a significant difference (*P* = 0.004). It is possible that pathological changes in small vessels in donor kidneys were directly related to donor age; arteriolopathy and microangiopathy possibly directly affect long-term function of renal grafts.

In the past 10 years, owing to serious deficiencies in sourcing kidneys, the age of marginal kidney donors has increased in all of the world. In current implemented expanded criteria for donor kidneys [14], the upper age limit for marginal healthy kidney donors without complications is determined as 60 years. Some investigations have reported that, in some cases, there has been no upper limit for a donor's age [15]. Studies have reported that long-term renal function of older donor renal grafts is much lower than that of younger donors. Despite such reports, the previous upper age limit of 50 years (which had been maintained for 30 years) has risen to around 65 years. It has been suggested that the reason for this increase is due to the status of elder donors in recent years. That is, they are generally healthier and pay closer attention to their lifestyle choices, and medical insurance coverage is now better after donation [16]. Researchers from the Tokyo Women's Medical University in Japan reported 242 donors with persistent hematuria and albuminuria after donation, with a mean age of 57 years [17]. The study reported that 8.3% of donors had albuminuria and 5.2% had microscopic hematuria after donation. The earliest abnormal urine test case occurred 3 months after donation. Di Cocco et al. summarized clinical data, such as blood pressure, body weight index, ischemic time, and accompanied complications, of 32 cases who received kidneys from donors aged over 60 years [18]. The conclusion reached by the researchers in that study is similar to that of the current study. That is, long-term renal function of kidneys from elder donors is significantly lower than long-term renal function of kidneys from younger donors. Time-Zero pathological results from the current study's cohort were 15 cases with obvious microangiopathy (mean age of 49 years), which accounted for 20.83% of all cases. It is therefore suggested that a cautious approach should be taken when considering expansion of donor age limits, with full consideration being given to subclinical pathological changes caused by age-related factors. Long-term renal function changes and survival status should be strictly monitored at the same time.

Previous research has reported that the serum Cr clearance rate decreases 7 mL/min·1.73 m every 10 years after a person reaches the age of 30 and renal blood flow decreases 10% every 10 years after a person reaches the age of 40 years [19]. Corresponding histological changes were glomerulosclerosis, tubular atrophy, and interstitial fibrosis. All of these micropathological changes could not be detected by noninvasive tests. Therefore, it is a physiological regularity that renal function would progressively deteriorate with increased age. Donor kidneys with such slight and hidden pathological changes could accelerate in their progression to clinical pathological status by ischemic injury, adverse effects of a drug, or immune reaction, for example, which could affect function and long-term survival of the renal graft. Surveys on Chinese chronic kidney disease by Zhang et al. found that, in several provinces of the China, for example, the incidence of chronic kidney disease in those aged older than 40 years reached 10.8% [20]. Although many people underwent a routine annual physical examination, the disease awareness rate was only 12.5% that could be explained partly by our data. Some patients are healthy kidney donor candidates but have hidden kidney diseases, which creates two potential hazards for the long-term renal function of recipients and the future healthy status of the donor.

This study had some limitations. The study was a retrospective case analysis, in which random subgroups and controls were lacking. In future studies, the methodology can be improved to be included in. As physical examination items are qualified for donors, this study was based on pathological data as the major evaluation criteria. Specific tests, such as immunofluorescence, immunohistochemistry, and electronic microscopy, should be used in future research but may not be able to be conducted on all participants owing to limitations in laboratory conditions and the economic status of patients.

## 5. Conclusions

The results of this study suggest that basic latent pathological changes, especially pathological changes in arteries of the donor kidney, could affect long-term renal function of the recipient, the extent of which is positively correlated with the pathological grade and scale. The age of donors may also be positively correlated with pathological changes. It is therefore suggested that doctors should give considerable thought to the age of the donor when deciding on marginal kidney donors. In particular, they should consider possible latent pathological changes due to the age of a donor. A Time-Zero biopsy can help doctors understand the status of the basic structure of the donor kidney and if any latent lesions may be present, especially for marginal kidney donation from an older donor. It may also assist with recovery prognosis of a renal graft after transplantation and long-term graft function. A Time-Zero biopsy may provide important evidence so doctors can accurately and confidently prescribe the correct immunosuppressive regimen for a patient and may assist doctors to provide reasonable advice to donors with regard to a healthy lifestyle over the long term following a donation. 

The results indicate that latent pathological changes that cannot be detected by routine noninvasive tests may exist in healthy populations, so that a cautious approach should be taken when considering expansion of donor age limit. This could possibly help explain the high incidence of kidney disease and the low disease awareness status in the general Chinese population.

## Figures and Tables

**Table 1:** Demographic data of donors and recipients.

Pathology	*n*	Parent donor (%)	FK/CSA*	Donor			Recipient		
				Age	M/F	BMI	Age	M/F	BMI
With path	37	10 (27.0%)	23/13	37 ± 8	22/15	22.53	35 ± 13	28/9	21.4
Without path	35	18 (51.43%)	15/19	44 ± 12	20/14	23.17	30 ± 10	27/7	22.5

*Triple immunosuppressive agent, basic FK, or CSA; BMI: body mass index; FK/CSA: tacrolimus/cyclosporin A; M/F: male/female.

**Table 2:** Pathological changes of donor kidney.

Pathological change	*n*	F/M	Age	D and R*
Capillary and arteriola	15	7/8	49 ± 9	Parents 9/sibling 3/cousins 3
Glomerulosclerosis	4	4/0	54 ± 3	Parents 3/sibling 1
Interstitial fibrosis	14	10/4	46 ± 12	Parents 7/sibling 4/cousins 3
Tubular injury**	16	11/5	43 ± 12	Parents 7/sibling 1/cousins 8

*D: donor, R: recipients, D and R relationship: a parent donated a kidney to a son or daughter, sibling: a sibling donated a kidney to their brother or sister. Other relationships were cousin-german relationships; **renal tubular injury: loss of the kidney tubule epithelial brush border, vacuolar, and granular degeneration of the tubular epithelium, tubular ectasia, and focal tubular atrophy. F/M: female/male.

**Table 3:** Comparisons between postoperative renal function in cases with or without pathological changes.

Group	Average age		SCr				BUN^&^				UA^Δ^			
	D	R	3**	6	12	24	3	6	12	24	3	6	12	24
Total	40 ± 7	33 ± 5	115 ± 26	120 ± 19	124 ± 13	138.8 ± 67	6.2 ± 1.6	6.6 ± 1.1	6.6 ± 1.8	7 ± 3.3	324 ± 91	350 ± 130	334 ± 13	349 ± 121
Normal	37 ± 8	35 ± 13	112 ± 18	106 ± 17	124.7 ± 19.8	113 ± 9.3	6.6 ± 1.4	6.4 ± 2.6	6.4 ± 3	6.4 ± 2.1	320 ± 46	329 ± 33	345 ± 37	344 ± 30
Change	44 ± 12	30 ± 10	111 ± 23	110 ± 19	109.6 ± 18	154 ± 140	7.1 ± 3.4	6.9 ± 1.9	6.7 ± 2	7.9 ± 3.6	336 ± 99	343 ± 87	42 ± 91	365 ± 98

Compared with and without pathological changes: *age of donor, *P* = 0.02; ^#^serum Cr (umol/L), *P* = 0.550; ^&^BUN (mmol/L), *P* = 0.13; ^Δ^uric acid, *P* = 0.11; **postoperative months; D: donor; R: recipient; UA: uric acid (mmol/L).

**Table 4:** Postoperative renal functions of patients who received a donor kidney with different pathological changes.

Path. change	*n* (%)	Average age		Serum Cr (umol/L)**				BUN (mmol/L)				Uric acid (mmol/L)			
		D	R	3 (mon)	6	12	24	3	6	12	24	3	6	12	24^&^
Normal	37 (51.39)	37 ± 8	35 ± 13	112 ± 18	106 ± 17	124.7 ± 19.8	113 ± 9.3	6.6 ± 1.4	6.4 ± 2.6	6.4 ± 3	6.4 ± 2.1	320 ± 46	329 ± 33	345 ± 37	344 ± 30
Tubule injury	22 (30.56)	44 ± 12	32 ± 11	113 ± 23	114 ± 18	112 ± 16.7	156 ± 144	7.9 ± 3.9	7.5 ± 2	7.1 ± 1.9	7.6 ± 3.8	334 ± 108	361 ± 83	376 ± 103	363 ± 90
Inte. fib.	15 (20.13)	46 ± 12	33 ± 10	109 ± 24	110 ± 20	111 ± 17	154 ± 171	7.2 ± 5	6.6 ± 2	6.7 ± 2	7.4 ± 4	366 ± 122	351 ± 88	46 ± 12	374 ± 98
GS	4 (5.56)	54 ± 3	31 ± 10	103 ± 17	98 ± 19	103 ± 13	270 ± 333	5.5 ± 1.1	6.9 ± 1.8	8.0 ± 1	10.9 ± 7.8	272 ± 43	321 ± 81	317 ± 72	336 ± 67
A pathy^#^	15 (20.83)	49 ± 9	29 ± 8	116 ± 20	112 ± 17	113.5 ± 13.2	208 ± 200	6.7 ± 2.1	7.0 ± 1.2	7.0 ± 1.5	9.6 ± 4.6	340 ± 79	340 ± 93	310 ± 54	366 ± 94

**Compared with normal, age, and serum Cr in 24 months were significantly different (*P* < 0.05) in GA and A. pathy; ^#^A: arteriolar pathology included degrees 1 and 2; compared with donor age, serum Cr were significantly different (*P* < 0.05). ^&^All groups of uric acid not different (*P* > 0.05).

**Table 5:** Long-term graft function in various arteriolopathy and microangiopathy compared with and without pathological changes.

Path. change	*n*	Average age*		Serum Cr (umol/L)^#^				BUN (mmol/L)^§^				Uric acid (mmol/L)^△^			
		D	R	3 (m)	6	12	24	3 (m)	6	12	24	3 (m)	6	12	24
Normal	37	37 ± 8	35 ± 13	112 ± 18	106 ± 17	124.7 ± 19.8	113 ± 9.3	6.6 ± 1.4	6.4 ± 2.6	6.4 ± 3	6.4 ± 2.1	320 ± 46	329 ± 33	345 ± 37	344 ± 30
Arteriolopathy I	9	46 ± 6	33 ± 9	119 ± 16	111 ± 9.8	113.5 ± 14.9	196 ± 268	7.1 ± 1.4	7.3 ± 1	7.2 ± 1.3	9.6 ± 6.2	360 ± 88	310 ± 77	329 ± 70	358 ± 91
Arteriolopathy II	6	53 ± 8	33 ± 9	113 ± 29	108 ± 21	112.3 ± 13.5	227 ± 192	6.1 ± 1.8	6.1 ± 0.7	6.5 ± 1.9	10 ± 4.4	310 ± 77	335 ± 106	282 ± 31	379 ± 126
Glomerulosclerosis	4^&^	54 ± 3	31 ± 10	103 ± 17	98 ± 19	103 ± 13	270 ± 333	5.0 ± 1.1	6.9 ± 1.8	8.0 ± 1	10.9 ± 7.8	272 ± 43	321 ± 81	317 ± 72	336 ± 67

Compared with normal: *age, *P* < 0.05; ^#^serum Cr at 24 months, *P* < 0.05; ^§^BUN at 24 months, *P* < 0.05; ^△^uric acid at 24 months, *P* > 0.05; ^&^three cases of complicated arteriolopathy were observed.
